# Rifaximin modulates TRH and TRH-like peptide expression throughout the brain and peripheral tissues of male rats

**DOI:** 10.1186/s12868-022-00694-z

**Published:** 2022-02-21

**Authors:** Albert Eugene Pekary, Albert Sattin

**Affiliations:** 1 Research Services, VA Greater Los Angeles Healthcare System, Bldg. 114, Rm. 229B, 11301 Wilshire Blvd., Los Angeles, CA 90073 USA; 2 Psychiatry Services, VA Greater Los Angeles Healthcare System, Los Angeles, CA 90073 USA; 3 Center for Ulcer Research and Education, VA Greater Los Angeles Healthcare System, Los Angeles, CA 90073 USA; 4 Departments of Psychiatry and Biobehavioral Sciences, University of California, Los Angeles, CA 90073 USA; 5 Brain Research Institute, University of California, CA 90073 Los Angeles, USA; 6 Department of Medicine, University of California, Los Angeles, CA 90073 USA

**Keywords:** TRH, Rifaximin, Medulla, Cortex, Prostate, Adrenal

## Abstract

**Background:**

The TRH/TRH-R1 receptor signaling pathway within the neurons of the dorsal vagal complex is an important mediator of the brain-gut axis. Mental health and protection from a variety of neuropathologies, such as autism, Attention Deficit Hyperactivity Disorder, Alzheimer’s and Parkinson’s disease, major depression, migraine and epilepsy are influenced by the gut microbiome and is mediated by the vagus nerve. The antibiotic rifaximin (RF) does not cross the gut-blood barrier. It changes the composition of the gut microbiome resulting in therapeutic benefits for traveler’s diarrhea, hepatic encephalopathy, and prostatitis. TRH and TRH-like peptides, with the structure pGlu-X-Pro-NH_2_, where “X” can be any amino acid residue, have reproduction-enhancing, caloric-restriction-like, anti-aging, pancreatic-β cell-, cardiovascular-, and neuroprotective effects. TRH and TRH-like peptides occur not only throughout the CNS but also in peripheral tissues. To elucidate the involvement of TRH-like peptides in brain-gut-reproductive system interactions 16 male Sprague–Dawley rats, 203 ± 6 g, were divided into 4 groups (n = 4/group): the control (CON) group remained on ad libitum Purina rodent chow and water for 10 days until decapitation, acute (AC) group receiving 150 mg RF/kg powdered rodent chow for 24 h providing 150 mg RF/kg body weight for 200 g rats, chronic (CHR) animals receiving RF for 10 days; withdrawal (WD) rats receiving RF for 8 days and then normal chow for 2 days.

**Results:**

Significant changes in the levels of TRH and TRH-like peptides occurred throughout the brain and peripheral tissues in response to RF. The number of significant changes in TRH and TRH-like peptide levels in brain resulting from RF treatment, in descending order were: medulla (16), piriform cortex (8), nucleus accumbens (7), frontal cortex (5), striatum (3), amygdala (3), entorhinal cortex (3), anterior (2), and posterior cingulate (2), hippocampus (1), hypothalamus (0) and cerebellum (0). The corresponding ranking for peripheral tissues were: prostate (6), adrenals (4), pancreas (3), liver (2), testis (1), heart (0).

**Conclusions:**

The sensitivity of TRH and TRH-like peptide expression to RF treatment, particularly in the medulla oblongata and prostate, is consistent with the participation of these peptides in the therapeutic effects of RF.

## Background

Mental health and protection from a variety of aging-related neurodegenerative disorders, such as autism, Attention Deficit Hyperactivity Disorder, Alzheimer’s and Parkinson’s disease, major depression, migraine and epilepsy, involve the gut microbiome and is mediated by the vagus nerve [1–4]. This is most evident in the behavioral abnormalities and GI symptoms of germ-free (GF) rodents [4, 5]. The mechanisms underlying these effects include reduced levels of brain-derived neurotrophic factors in the cortex, hippocampus, and amygdala, and altered expression of genes encoding subunits of the glutamate and dopamine receptors [1]. Glutamatergic neurons are the most abundant excitatory class of nerves in the mammalian nervous system which requires co-release  of the neuromodulatory thyrotropin releasing hormone (TRH) and TRH-like peptides to protect postsynaptic cells from the excitotoxic effects of excessive glutamate release [6–9]. The TRH/TRH-R1 receptor signaling pathway is an important mediator of brain-gut axis communication via the brain medulla oblongata and its associated TRH synthesizing neurons within the raphe pallidus, raphe obscura, and parapyramidal regions [10]. TRH and TRH-like peptides, with the structure pGlu-X-Pro-NH_2_ where “X” can be any amino acid residue, have reproductive, antidepressant, anxiolytic, analeptic, anorexic, and anti-aging effects [11].

TRH and TRH-like peptides occur not only throughout the CNS but also peripheral tissues, with very high levels in the rat and human prostate [11]. This is particularly noteworthy given the vulnerability of humans to prostatitis and prostate cancer [12]. Recent studies have implicated bacterial infections as potential causes of prostate diseases. The antibiotic rifaximin (RF), which does not cross the gut-blood barrier, is a standard treatment for traveler’s diarrhea and hepatic encephalopathy. Its therapeutic potential in the treatment of other brain and urogenital disorders is currently being evaluated [12].

RF has anti-depressant and anxiolytic effects in both humans and rodents which are mediated, at least in part, by its ability to modify the composition of the gut microbiota [13–18]. Inadequate and/or irregulate sleep and poor nutrition contribute to obesity, alterations in the microbiome and the expression of gut hormones, including leptin and ghrelin, which have a profound effect on both appetite [19–23] and TRH and TRH-like peptide release [24, 25]. Leptin and ghrelin also have mood altering effects [24, 25].

The present studies examine the effects of oral RF on TRH and TRH-like peptide levels in those brain regions, for example the medulla oblongata, and peripheral tissues which may play a role in the therapeutic effects of this gut-limited antibiotic [12].

## Methods

### Animals

“Young adult male Sprague–Dawley rats (n = 16, SPF, Envigo, Indianapolis, IN) were used for all experiments. These animals were group housed (2 animals per cage) on wood shavings with a red plastic tube for play and shelter. Standard Purina rodent chow #5001 and water were provided ad libitum during a standard one-week initial quarantine with 22 ± 2 °C and 50 ± 10% relative humidity; lights on: 6 am–6 pm. Cages, water and bedding were changed every 3 days. All animals were weighed on the day of receipt and on the morning of each experiment. Initial body weights did not differ between experimental groups. Animals were randomized prior to the start of rifaximin treatment. Research was approved by the VA Greater Los Angeles Healthcare System Animal Care and Use Committee (IACUC Protocol #030090-10) and conducted in compliance with the Animal Welfare Act and the federal statutes and regulations related to animals and experiments involving animals and adheres to principles stated in the Guide for the Care and use of Laboratory Animals, Eighth Edition, NRC Publication, 2011. All efforts have been made to minimize the number of animals used and their suffering. Animal was handled for 10 min per day for one month and then transferred from the Veterinary Medical Unit to the laboratory 12 h before the start of experiments to minimize the stress of a novel environment” [11]. “The American Veterinary Medical Association has concluded that decapitation without prior sedation ‘is conditionally acceptable if performed correctly, and it should be used in research settings when its use is required by the experimental design and approved by the Institutional Animal Care and Use Committee’” [26]. This study is reported in accordance with ARRIVE guidelines (Animal Research: Reporting of In Vivo Experiments) (https://arriveguidelines.org).

“Because of the 10- to 100-fold changes in TRH and TRH-like peptide levels in response to the estrus cycle. female rats were not included in the present study” [27].

### Effect of acute, chronic and withdrawal treatment with rifaximin in normal rat chow on levels of TRH and TRH-like peptides in rat brain and peripheral tissues

Sixteen young adult male Sprague–Dawley rats, body weight (mean ± SD) 203 ± 6 g, 3.0% CV, were divided into 4 groups (n = 4/group). The control (CON) group remained on ad libitum standard Purina powdered rodent chow and water for 10 days until decapitation. The acute (AC) group received ad libitum powdered rodent chow and water for 9 days and then 1 g rifaximin (Sigma, St. Louis, MO)/500 g powdered rat chow for 24 h. Assuming 16.7 g chow consumption/day, this would provide 150 mg rifaximin/kg body weight for 200 g rats. The chronic (CHR) animals received RF in powdered chow for 10 days. The withdrawal (WD) rats received RF chow for 8 days and then normal chow for 2 days. The effect of RF withdrawal on TRH and TRH-like peptide levels when compared to the corresponding acute effects can reveal the relative contribution of changes in peptide biosynthesis (hours) to changes in peptide release (minutes) [28].

### Dissection of rat brain and peripheral tissues

All rats were decapitated without anesthesia to avoid rapid, anesthetic-induced, blockade of peptide release [29]. Nucleus accumbens (NA), amygdala (AY), frontal cortex (FCX), cerebellum (CBL), medulla oblongata (MED), anterior cingulate (ACNG), posterior cingulate (PCNG), striatum (STR), piriform cortex (PIR), hippocampus (HC), entorhinal cortex (ENT), adrenals (AD), pancreas (PAN), prostate (PR), epididymis (EP), testis (T), heart (H) and liver (L) were hand dissected, weighed rapidly, and then extracted as previously described in detail [30].

### Serum hormone assays

Serum rat leptin, rat insulin, testosterone, free T_4_, total T_3_ and glucose were measured (assay range, intra-assay CV%) with the following commercial RIA kits: rat leptin (0.801–200 ng/ml, 3.2) and rat insulin (0.0329–2.0 ng/ml, 4.8) (Linco Research, Inc., St. Charles, MO), testosterone (0.05–40 ng/ml, 6.7), free T_4_ (0.045–60 ng/DL, 4.6) and total T_3_ (0.06–80 pg/ml, 4.8) (MP Biomedical, Solon, OH). Serum glucose was measured with the Contour Next EZ Blood Glucose Monitoring System (Ascensia Diabetes Care US, Inc., Parsippany, NJ).

### HPLC and RIA procedures, HPLC peak identification and quantitation

HPLC and RIA procedures, peak identification, and quantitation by co-chromatography with synthetic TRH and TRH-like peptides, relative potency analysis of multiple antibodies to TRH and TRH-like peptides, and mass spectrometry and have been previously reported in detail [11, 28, 31–33].

**Fig. 1:**
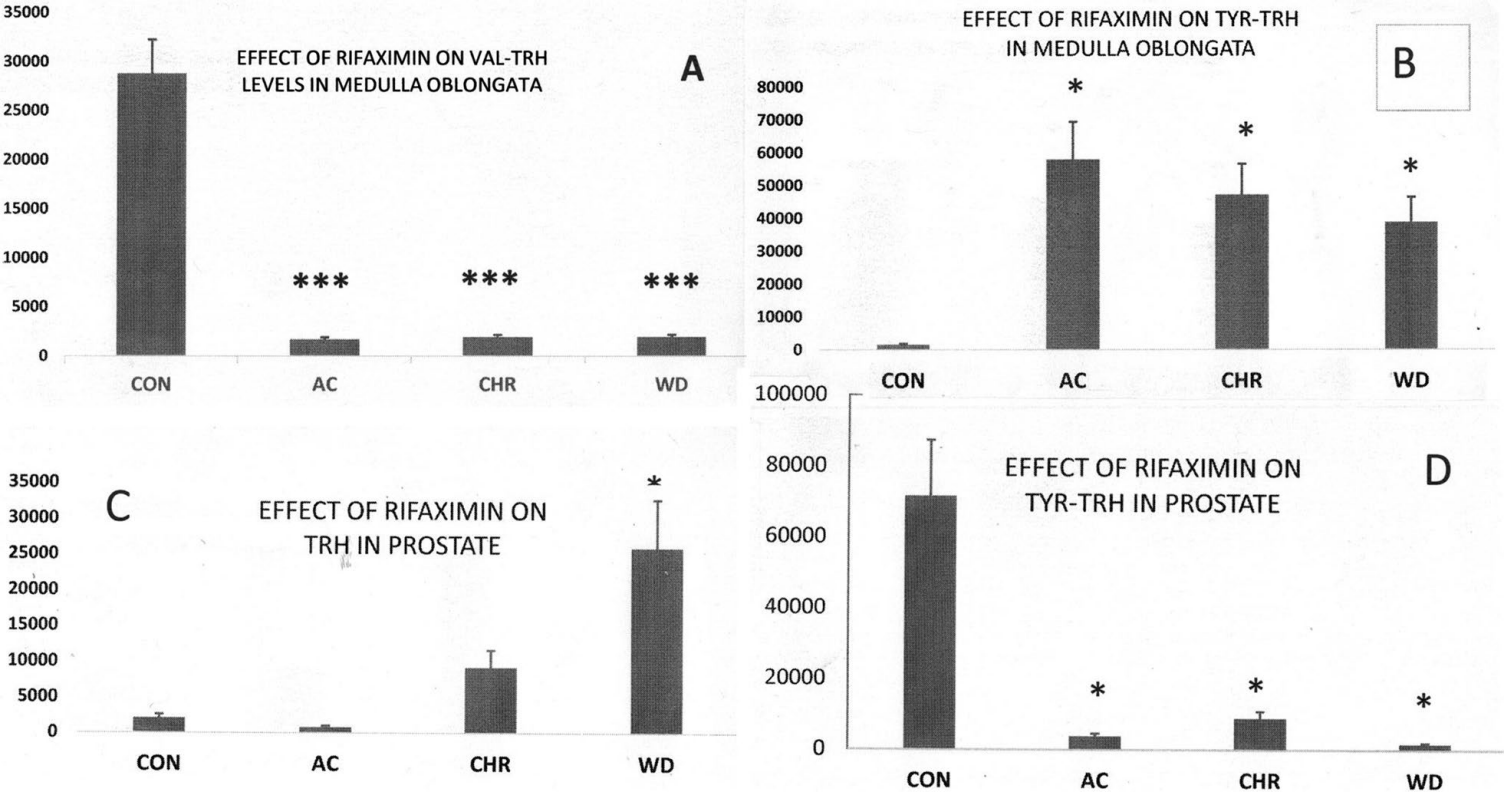
Representative profiles of TRH and TRH-like peptide responses in male rats to RF treatment. The response patterns in **A** and **D** are consistent with rapid and sustained increase in peptide release (reduced peptide level). The profile in **B** suggests rapid and sustained decrease in peptide release during and after RF exposure. **C** could be explained by RF stimulation of sustained peptide release which is compensated by increased peptide synthesis. Withdrawal of RF reduced peptide release but the compensatory increase in peptide synthesis results in a rebound increase in peptide content. The persistence of changes in TRH and TRH-like peptide levels in the WD group after RF has completely cleared from the GI tract is consistent with lingering effects of an altered microbiome

Briefly, after boiling, tissues were dried, re-extracted with methanol, dried and defatted by water—ethyl ether partitioning. Dried samples were dissolved in 0.1%trifluroacetic acid (TFA) and loaded onto reverse phaseC18 Sep-Pak cartridges (Water, Milford, MA). TRH and TRH-like peptides were eluted with 50% methanol. Dried peptides were again dissolved in TFA, filtered and then fractionated by HPLC using a 4.6–150 mm Econosphere, 3 mm C18 reverse phase column (Dr. Maisch GmbH, Ammerbuch, Germany) and a 0.2%/min gradient of acetonitrile. The 0.5 ml fractions collected were dried completely and reconstituted with 0.10 ml of 0.02% NaN3 just before RIA (Fig. 1).

The antiserum used (8B9) cross-reacts with TRH and nine TRH-like peptides with a relative potency of displacement ranging from 2.31 (Lys-TRH) to 0.288 (Ser-TRH) relative to Tyr-TRH (Table 2), (see [28]). Two of the regularly observed peaks (2a, b) consist of a mixture of unidentified TRH-like peptides. Of the eight observed peptides three have so far been confirmed by mass spectrometry: TRH, Glu-TRH and Tyr-TRH [31]. Tissue samples from the 4 rats within each treatment group were pooled prior to HPLC to provide the minimum amount of immunoreactivity needed for reliable RIA measurements.

The mean recovery of TRH and TRH-like peptide immunoreactivity from all tissues studied was 84 ± 15% (mean ± SD). The within-assay and between-assay coefficient of variation for measuring 333 pg/ml TRH was 4.8% and 16.9%, respectively. All HPLC fractions obtained from a given brain region or peripheral tissue were analyzed in the same RIA. The minimum detectable dose for TRH was 5 pg/ml. The specific binding of [^125^I]TRH (Bo/T) was 25%.

### Statistical analysis

“Statistical methods for comparing peak areas were made with the aid of Statview (Abacus Concepts, Inc., Berkeley, CA), a statistical software package for the Macintosh computer. All multi-group comparisons were carried out by one way analysis of variance using post hoc Scheffe contrast with the control group” [30].

“The mean within-group coefficient of variation (CV) (SD/mean, CV-within group) for each tissue and TRH/TRH-like peptide combination, across four photoperiod intervals, has been previously reported (circadian rhythm experiment) for untreated Sprague–Dawley male rats” [30]. Mean within-group CVs in brain ranged from 4.5% for TRH levels in AY to 43% for Phe-TRH in HY, and from 12% for Val-TRH in testis to 41% for Trp-TRH in EP for peripheral tissues. These CVs were then used to estimate the level of significance, by on way ANOVA, of changes in the pooled mean values (see [34]) of TRH and TRH-like peptide levels following acute (AC), chronic (CHR) and withdrawal (WD) ingestion of RF” [30]. Pooling of at least 4 tissue extracts was required to provide sufficient signal-to-noise in the RIA for many brain regions and to keep the total number of HPLC fractions to be analyzed reasonable: 4 treatment groups × 19 tissues × 100 HPLC fractions/tissue pool = 7600 RIA samples for the present study. Without pooling the total number of HPLC fractions would have been 4 × 7600 = 30,400.

## Results

### Body weights

Mean body weights for all animals at the time of decapitation (9 weeks) was 269 ± 12 g, 4.5% CV. Mean animal weights for each RF treatment group did not differ significantly with the untreated controls by one way ANOVA.

### Serum hormone levels following oral rifaximin

Serum glucose levels for the CHR group were significantly lowers than the WD group (p < 0.05). All other serum hormone levels did not differ significantly between experimental groups by one way ANOVA (Table 1).

**Table 1:** Effect of oral rifaximin on serum hormone levels of male rats

	Testosterone nmol/L	fT_3_ pg/ml	fT_4_ ng/dl	Leptin ng/ml	Rat insulin ng/ml	Glucose mg/dl
CON	16.8 ± 9.1	2.61 ± 0.30	2.94 ± 0.24	3.97 ± 1.27	0.15 ± 0.04	130 ± 8
AC	12.3 ± 4.8	1.90 ± 0.35	2.69 ± 0.49	3.69 ± 1.88	0.15 ± 0.09	128 ± 19
CHR	15.5 ± 4.2	2.43 ± 0.45	2.72 ± 0.19	4.43 ± 1.06	0.21 ± 0.04	123 ± 4*
WD	14.8 ± 8.0	2.54 ± 0.46	3.14 ± 0.37	2.95 ± 0.51	0.30 ± 0.18	145 ± 16

There were no significant changes by one way ANOVA versus the corresponding control group. All results are mean ± SD

^*^p < 0.05 by one-way ANOVA versus the WD group

## Overview of TRH and TRH-like peptide data selection and presentation

Our combined HPLC-RIA methodology can resolve 10 TRH and TRH-like peptides: Glu-TRH, Peaks 2a, b (partially resolved mixture of TRH-like peptides), TRH, Val-TRH, Thr-TRH, Tyr-TRH, Leu-TRH, Phe-TRH and Trp-TRH [35]. The present study evaluated 12 brain regions and 7 peripheral tissues. This represents 10 × 19 = 190 peptide mean values.

### HPLC results in brain and peripheral tissues

Significant, 25- to 37-fold, increases in Tyr-TRH and 93–94% decreases in Val-TRH levels in medulla oblongata (all treatment groups, Table 2) and increases in TRH (13-fold, WD group) and Val-TRH (fivefold, WD group) and 99% decreases in Tyr-TRH concentrations in ventral prostate and a 36-fold increase in liver Tyr-TRH (WD group) (Table 3) were observed following rifaximin administration.

**Table 2:** Effect of acute (AC), chronic (CHR) and withdrawal (WD) treatments with oral rifaximin on TRH and TRH-like peptide levels in brain regions of male rats (pg)

Frontal cortex	Glu-TRH	Peak 2	TRH	Val-TRH	Tyr-TRH	Leu-TRH	Phe-TRH	Trp-TRH
CON	80 ± 16	815 ± 156	1954 ± 207	1496 ± 307	3556 ± 580	1857 ± 696	1924 ± 327	736 ± 161
AC	494 ± 101**	945 ± 180	1598 ± 169	876 ± 180	1577 ± 257*	868 ± 326	1132 ± 192	521 ± 114
CHR	188 ± 39	1143 ± 218	1867 ± 198	1485 ± 304	4283 ± 698	1761 ± 660	1487 ± 253	837 ± 183
WD	395 ± 81*	874 ± 167	1310 ± 139	285 ± 59*	1515 ± 247*	904 ± 339	1087 ± 185	369 ± 81
Hypothalamus								
CON	657 ± 217	1771 ± 638	38,509 ± 12,323	0	0	1706 ± 699	999 ± 430	616 ± 172
AC	638 ± 211	1690 ± 608	34,863 ± 11,156	0	0	1368 ± 561	1138 ± 489	436 ± 122
CHR	644 ± 213	1914 ± 689	41,487 ± 13,276	0	0	1811 ± 743	943 ± 405	371 ± 104
WD	841 ± 278	3983 ± 1434	28,634 ± 9163	0	0	1549 ± 635	1104 ± 475	915 ± 256
Amygdala								
CON	597 ± 169	1347 ± 180	513 ± 23	1312 ± 125	1543 ± 262	8408 ± 2262	1591 ± 248	1344 ± 285
AC	324 ± 92	575 ± 77	1055 ± 47	980 ± 93	830 ± 141	7736 ± 2081	896 ± 140	378 ± 80*
CHR	782 ± 221	467 ± 63	1580 ± 71*	1234 ± 117	685 ± 116	9188 ± 2472	1961 ± 306	1131 ± 240
WD	410 ± 116	284 ± 38*	1012 ± 46	757 ± 72	1245 ± 212	5476 ± 1473	632 ± 99	973 ± 206
Hippocampus								
CON	183 ± 34	1959 ± 402	2561 ± 597	1248 ± 124	3691 ± 993	957 ± 257	2457 ± 383	1251 ± 133
AC	562 ± 103*	1097 ± 225	1349 ± 314	808 ± 80	4410 ± 1186	1536 ± 413	1188 ± 185	690 ± 73
CHR	266 ± 49	1545 ± 317	2023 ± 471	1821 ± 180	4980 ± 1340	1930 ± 519	1343 ± 210	1167 ± 124
WD	103 ± 19	1033 ± 212	1842 ± 429	1761 ± 174	8267 ± 2224	2000 ± 538	1516 ± 236	1379 ± 146
Piriform cortex								
CON	609 ± 121	860 ± 127	1324 ± 207	1100 ± 155	4284 ± 878	1576 ± 345	1856 ± 380	1203 ± 383
AC	360 ± 71	1464 ± 217	0	2872 ± 405*	1055 ± 216*	822 ± 180	592 ± 121*	256 ± 81*
CHR	131 ± 26*	515 ± 76	1104 ± 172	584 ± 82	2821 ± 578	824 ± 180	865 ± 177	359 ± 114*
WD	235 ± 47	176 ± 26	1268 ± 198	888 ± 125	2921 ± 599	967 ± 212	610 ± 125*	203 ± 65*
Nucleus accumbens								
CON	787 ± 83	3731 ± 302	17,232 ± 2188	0	0	1385 ± 392	1437 ± 162	583 ± 111
AC	506 ± 54	1673 ± 136	12,653 ± 1607	0	0	948 ± 268	768 ± 87	300 ± 57
CHR	394 ± 42	1304 ± 106*	5570 ± 707*	0	0	861 ± 244	627 ± 71	445 ± 85
WD	88 ± 9**	486 ± 39*	3792 ± 482*	0	0	162 ± 46	207 ± 23*	95 ± 18*
Entorhinal cortex								
CON	63 ± 13	436 ± 38	903 ± 121	652 ± 83	3511 ± 572	1497 ± 222	1461 ± 346	581 ± 86
AC	214 ± 45	657 ± 58	749 ± 100	803 ± 102	1770 ± 289	816 ± 121	656 ± 155	397 ± 59
CHR	244 ± 52	1275 ± 112*	2017 ± 270	1052 ± 134	3271 ± 533	1913 ± 283	1052 ± 249	1005 ± 149
WD	312 ± 66*	1269 ± 112*	1613 ± 216	1661 ± 211	6523 ± 1063	1763 ± 261	1424 ± 337	940 ± 139
Striatum								
CON	1724 ± 207	1992 ± 294	667 ± 66	1448 ± 226	983 ± 153	9623 ± 1155	2316 ± 113	2363 ± 250
AC	534 ± 64	1565 ± 232	1522 ± 151	1280 ± 200	2642 ± 412	9292 ± 1115	1071 ± 52	1373 ± 145
CHR	2718 ± 326	401 ± 59*	1781 ± 176	764 ± 119	2205 ± 344	14,840 ± 1781	2720 ± 133	1459 ± 155
WD	2091 ± 251	717 ± 106*	2916 ± 289*	1983 ± 309	1227 ± 191	14,663 ± 1760	2086 ± 102	2154 ± 228
Medulla oblongata								
CON	2187 ± 372	492 ± 45	5377 ± 645	28,807 ± 3860	1555 ± 308	1908 ± 500	12,443 ± 2551	2087 ± 399
AC	3269 ± 556	347 ± 32	2263 ± 272*	1709 ± 229***	58,005 ± 11,485*	2335 ± 612	2087 ± 428**	915 ± 175*
CHR	4269 ± 726	1562 ± 144*	1293 ± 155**	1938 ± 260***	47,265 ± 9359*	2390 ± 626	2133 ± 437**	715 ± 137*
WD	1916 ± 326	172 ± 16	1550 ± 186**	1967 ± 264***	38,788 ± 7680*	1147 ± 301	1312 ± 269**	355 ± 68**
Cerebellum								
CON	1636 ± 128	332 ± 24	3747 ± 502	2328 ± 247	11,040 ± 1557	3041 ± 988	2845 ± 321	2064 ± 584
AC	1539 ± 120	559 ± 40	2461 ± 330	2118 ± 225	10,117 ± 1426	2070 ± 673	1881 ± 213	1004 ± 284
CHR	–	–	–	–	–	–	–	–
WD	2200 ± 172	1020 ± 72	4710 ± 631	2070 ± 219	10,320 ± 1455	2220 ± 722	5660 ± 640	1480 ± 419

Anterior	Cingulate							
CON	313 ± 31	2352 ± 315	684 ± 150	579 ± 115	1769 ± 276	993 ± 337	997 ± 254	385 ± 76
AC	178 ± 18	545 ± 73*	895 ± 196	1055 ± 209	1945 ± 303	666 ± 226	544 ± 139	240 ± 48
CHR	202 ± 20	776 ± 104	1076 ± 236	750 ± 149	2009 ± 313	722 ± 245	934 ± 238	586 ± 116
WD	207 ± 20	398 ± 53**	700 ± 153	536 ± 106	1387 ± 216	200 ± 68	663 ± 169	223 ± 44

Posterior	Cingulate							
CON	27 ± 6	521 ± 100	1967 ± 209	912 ± 187	4697 ± 766	1676 ± 629	1217 ± 207	353 ± 77
AC	138 ± 28*	944 ± 180	1319 ± 140	885 ± 181	3286 ± 536	976 ± 366	950 ± 162	943 ± 207
CHR	197 ± 40*	1486 ± 284	1659 ± 176	1093 ± 224	4470 ± 729	1465 ± 549	1151 ± 196	753 ± 165
WD	116 ± 24	1403 ± 268	1616 ± 172	1818 ± 373	13,661 ± 2227	1776 ± 666	1870 ± 318	676 ± 148

All results are mean ± SD

^*^p < 0.05; **p < 0.01; ***p < 0.002 by one way ANOVA using post hoc Scheffe contrasts versus the control group

**Table 3:** Effect of acute (AC), chronic (CHR) and withdrawal (WD) treatments with rifaximin on TRH and TRH-like peptide levels in peripheral tissues (pg)

Prostate	Glu-TRH	Peak 2	TRH	Val-TRH	Tyr-TRH	Leu-TRH	Phe-TRH	Trp-TRH
CON	1898 ± 645	48,829 ± 15,625	2068 ± 765	12,830 ± 3464	71,618 ± 22,202	9626 ± 3562	9435 ± 2925	4285 ± 1328
AC	1993 ± 678	39,195 ± 12,542	800 ± 296	8922 ± 2409	3748 ± 1162*	13,415 ± 4964	47,016 ± 14,575*	2981 ± 924
CHR	1827 ± 621	33,166 ± 10,613	9192 ± 3401	41,357 ± 11,166	8858 ± 2746*	4079 ± 1509	8103 ± 2512	3971 ± 1231
WD	3215 ± 1093	54,959 ± 17,587	25,935 ± 9596*	64,372 ± 17,380*	1640 ± 508*	4200 ± 1554	10,188 ± 3158	4346 ± 1347
Liver								
CON	277 ± 66	216 ± 41	584 ± 228	361 ± 105	58 ± 17	765 ± 245	452 ± 145	327 ± 75
AC	426 ± 102	313 ± 59	954 ± 372	394 ± 114	36 ± 11	1390 ± 445	930 ± 298	708 ± 163
CHR	586 ± 141	385 ± 73	1919 ± 748	2050 ± 595*	230 ± 69	537 ± 172	923 ± 295	360 ± 83
WD	626 ± 150	224 ± 43	1427 ± 557	738 ± 214	2073 ± 622*	571 ± 183	1167 ± 373	451 ± 104
Testis								
CON	43 ± 10	260 ± 99	714 ± 150	448 ± 54	2228 ± 646	656 ± 190	610 ± 140	174 ± 52
AC	93 ± 21	68 ± 26	370 ± 78	515 ± 62	2343 ± 679	807 ± 234	623 ± 143	448 ± 134
CHR	275 ± 63*	218 ± 83	693 ± 146	716 ± 86	3005 ± 871	957 ± 278	1190 ± 274	521 ± 156
WD	83 ± 19	160 ± 61	392 ± 82	460 ± 55	961 ± 279	516 ± 150	577 ± 133	442 ± 133
Heart								
CON	181 ± 54	134 ± 24	427 ± 51	408 ± 126	104 ± 31	533 ± 139	575 ± 196	271 ± 98
AC	170 ± 51	86 ± 15	242 ± 29	114 ± 35	159 ± 48	233 ± 61	247 ± 84	195 ± 70
CHR	123 ± 37	102 ± 18	271 ± 33	344 ± 107	73 ± 22	304 ± 79	776 ± 264	415 ± 149
WD	381 ± 114	203 ± 37	312 ± 37	137 ± 42	283 ± 85	227 ± 59	422 ± 143	307 ± 111
Pancreas								
CON	234 ± 82	279 ± 95	139 ± 58	206 ± 47	214 ± 45	870 ± 200	464 ± 97	531 ± 117
AC	191 ± 67	243 ± 83	115 ± 48	175 ± 40	165 ± 35	503 ± 116	419 ± 88	319 ± 70
CHR	101 ± 35	313 ± 106	173 ± 73	168 ± 39	114 ± 24	508 ± 117	1085 ± 228*	620 ± 136
WD	344 ± 120	238 ± 81	64 ± 27	75 ± 17	80 ± 17*	241 ± 55*	398 ± 84	523 ± 115
Adrenals								
CON	588 ± 188	1905 ± 286	1577 ± 315	1169 ± 339	2109 ± 970	1432 ± 473	1877 ± 507	1101 ± 374
AC	484 ± 155	1194 ± 179	856 ± 171	934 ± 271	1641 ± 755	535 ± 177	1288 ± 348	1145 ± 389
CHR	403 ± 129	835 ± 125*	871 ± 174	564 ± 164	1646 ± 757	616 ± 203	701 ± 189	216 ± 73
WD	74 ± 24	124 ± 19**	692 ± 138*	523 ± 152	631 ± 290	198 ± 65*	448 ± 121	124 ± 42

All results are mean ± SD

^*^p < 0.05; **p < 0.01 by one way ANOVA using post hoc Scheffe contrasts versus the control group

The number of significant changes in TRH and TRH-like peptide levels in brain resulting from RF treatment (In parentheses), in descending order were: MED (16), PIR (8), NA (7), FCX (5), STR (3), AY (3), ENT (3), ACNG (2), PCNG (2), HC (1), HY (0) and CBL (0) as seen in Table 2. The corresponding ranking for peripheral tissues were: PR (6), AD (4), PAN (3), L (2), T (1), H (0), (see Table 3). The pooled EP controls were lost during extraction so results for this tissue could not be analyzed.

## Discussion

Acute, chronic and withdrawal treatment with RF resulted in significant decreases in TRH, Val-TRH, Phe-TRH and Trp-TRH and marked increases in Tyr-TRH levels in the MED (Table 2). These changes result from alterations in the biosynthesis and release of these tripeptides. The rapidity of these responses is consistent with increased TRH, Val-TRH, Phe-TRH and Trp-TRH and decreased Tyr-TRH release, respectively [32]. These remarkable changes in peptide levels within the MED is consistent with current knowledge regarding the role of TRH (and TRH-like peptides) as mediators of brain-gut communication via the vagus nerve [10, 36]. The antidepressant activity of Tyr-TRH [31] and analeptic effect of Val-TRH [37] correspond with actions of TRH [11]. TRH and TRH-like peptide biosynthesis occurs within large dense core vesicles (LDCV) of glutamatergic neurons [32, 33]. They are co-released with glutamate and act to moderate the effects of this excitotoxic neurotransmitter [7, 8]. Neuropeptides, such as TRH, which are co-released with classical neurotransmitters are now considered primary mediators of brain circuit connectivity with a longer duration of action [38].

Dysbiosis of the microbiome has been implicated in prostatitis and prostate cancer [12, 39]. Rifaximin, an antibiotic which does not cross the blood-gut barrier, is currently being evaluated as a treatment for these pathologies [40]. It is noteworthy that among the peripheral tissues analyzed, PR had the highest number of significant changes in TRH and TRH-like peptide levels (Table 3) in response to RF treatment. PR has very high levels of TRH and TRH-like peptides which are subject to marked circadian rhythmicity [11]. TRH stimulates the adenylyl cyclase in basal cell membranes of the rat ventral prostate [41].

Withdrawal of RF increased TRH and Val-TRH levels in prostate (Table 3), which is consistent with RF stimulation of both biosynthesis and release of these peptides. Because the reduction in the peptide release rate is rapid but the changes in RF-stimulated peptide synthesis is slow with RF withdrawal, a rebound in the levels of these peptides is observed. Acute RF treatment increased Phe-TRH levels consistent with a rapid inhibition of release for this peptide in response to RF followed by a compensatory decrease in biosynthesis returning CHR Phe-TRH levels to CON values. AC, CHR and WD treatment with RF all decreased Tyr-TRH levels significantly in prostate (Table 3). These observations may reveal a rapid and sustained increase in Tyr-TRH release in response to RF treatment which is not accompanied by marked changes in the processing of Tyr-TRH progenitor peptides.

Withdrawal of RF resulted in significant decreases in the levels of all TRH and TRH-like peptides measured in the adrenals (Table 3). This is consistent with an acute decrease in biosynthesis and/or increase in release of these peptides [32]. RF has modest, transient, and beneficial effects on stress-related changes in the gut microbiome, inflammation, permeability and hyperalgesia as well as central responses to social stress [15, 17, 18, 42]. Manipulation of the gut microbiome can have significant effects on cortisol levels in urine [1]. The influence of the microbiota on the function of the HPA axis which regulates corticosterone levels was first demonstrated by Sudo et al. [43]. Germ-free mice have reduced levels of brain-derived neurotrophic factors in the cortex, hippocampus, and amygdala, and altered expression of glutamate and dopamine receptors in specific regions of the brain [44].

Administration of the probiotic bacterium *Lactobacillus rhamnosus* strain JB-1™ to mice significantly altered the expression of the gene coding for a GABA receptor in multiple regions of the brain, including the amygdala, hippocampus, and cortex. This effect was abolished by vagotomy [45].

The vagus nerve is the principal neuronal link between internal organs and the brain and has now been shown to be integral to the regulation of an array of autonomic functions, such as breathing, heart functions, pancreatic and liver regulation of metabolism, modulation of immune and inflammatory responses via the spleen, mood, and even consciousness [46, 47].

The TRH-degrading serum enzyme is a product of liver [48]. It rapidly metabolizes TRH and most TRH-like peptides in the circulation except Glu-TRH [49]. Rifaximin is used for the prevention of recurrent overt hepatic encephalopathy [50]. Significant increases in Val-TRH and Tyr-TRH levels were observed in liver in response to RF treatment (Table 3). Functional brain MRI studies of the responses of patients with cirrhosis to rifaximin treatment reveal higher activation in various brain regions including the frontal cortex, hippocampus, anterior and posterior cingulate [51]. RF is also utilized for the treatment of irritable bowel disease, diverticular disease, and small bowel bacterial overgrowth [16, 17, 50]. RF modulates inflammatory cytokines and intestinal permeability [52]. Medullary TRH and gastric vagal efferent and afferent circuits play a crucial role in the modulation of gastric integrity [53].

## Conclusions

The marked responsivity of TRH and TRH-like peptide expression to RF-induced alterations in gut microbiota of normal rats is consistent with the participation of these peptides in vagally-mediated brain-gut signaling. The observed effects persist after RF, which does not cross the blood-gut barrier, has cleared the GI tract. We expect future studies will extend this concept to antidepressant, anxiolytic, anti-obesity, GI-, liver- and prostate-protective effects of rifaximin [54].

## Data Availability

All statistically summarized data are included in this published article. Primary data available from AEP upon reasonable request.
